# Supplementary material for: Species composition and distribution of the Anopheles gambiae complex circulating in Kinshasa
Source: GigaByte. 2024 Jan 3;2024:gigabyte104. doi: 10.46471/gigabyte.104 (PMC10777374; doi:10.46471/gigabyte.104)
Supplement: Translation

# Translation: Composition et distribution des espèces du complexe *Anopheles gambiae* circulant à Kinshasa

**Authors:** Josue Zanga, Emery Metelo, Nono Mvuama, Victoire Nsabatien, Vanessa Mvudi, Degani Banzulu, Osée Mansiangi, Maxwel Bamba, Narcisse Basosila, Rodrigue Agossa, Roger Wumba

**Affiliations:** ^1^ Université de Kinshasa, Département de médecine tropicale, Kinshasa, République démocratique du Congo; ^2^ École de santé publique de Kinshasa, Laboratoire de bio-écologie et lutte antivectorielle, Département Santé et Environnement, Kinshasa, République démocratique du Congo; ^3^ Université de Bandundu, Faculté de médecine, Bandundu Ville, République démocratique du Congo; ^4^ Laboratoire de Bioécologie et Lutte Vectorielle, Département de Santé Environnementale, École de Santé Publique de Kinshasa, Kinshasa, République démocratique du Congo; ^5^ Université de Kinshasa, département de neurologie, Kinshasa, République démocratique du Congo; ^6^ Programme national de lutte contre le paludisme, Service de contrôle des vecteurs, Kinshasa, République démocratique du Congo; ^7^ Centre de recherche entomologique de Cotonou (CREC), Cotonou, Bénin

## Abstract

Il est essentiel de comprendre la distribution des espèces anophélienne pour planifier et mettre en œuvre des programmes de lutte contre le paludisme. Cette étude a évalué la composition et la distribution des espèces cryptiques du principal vecteur du paludisme, le complexe *Anopheles gambiae*, dans différents districts de Kinshasa. Des anophèles ont été échantillonnés à l’aide de pièges lumineux CDC dans les quatre districts de Kinshasa entre juillet 2021 et juin 2022, puis identifiés morphologiquement. Des proportions égales de *Anopheles gambiae* s.l. par site ont été soumises à une réaction en chaîne par polymérase pour identifier les espèces cryptiques du complexe *Anopheles gambiae*. Des spécimens du complexe *Anopheles gambiae* ont été identifiés dans tout Kinshasa. La densité moyenne diffère significativement à l’intérieur et à l’extérieur des ménages. Deux espèces de ce complexe circulent à Kinshasa : *Anopheles gambiae* et *Anopheles coluzzii*. Dans tous les sites étudiés, *Anopheles gambiae* était l’espèce la plus répandue. Nos résultats fournissent une base importante pour de futures études sur l’écologie et la dynamique des espèces cryptiques du complexe *Anopheles gambiae* à Kinshasa.

## Description des données

### Historique et contexte

Le genre *Anopheles* est de loin le vecteur Culicidae le plus ciblé par les efforts de lutte antivectorielle. Ce vecteur est responsable de la transmission du paludisme, une maladie infectieuse causée par des parasites protozoaires du genre *Plasmodium*, transmis par des moustiques *Anophèles* femelles [1.]. Sur les 465 espèces de moustiques *Anophèles* officiellement reconnues, environ 70 peuvent transmettre des parasites du paludisme à l’homme [2.]. En Afrique, les principaux vecteurs du paludisme appartiennent à quatre catégories taxonomiques : le complexe *Anopheles gambiae*, *Anopheles funestus* gpe, *Anopheles nili* gpe et *Anopheles moucheti* gpe [3., 4.]. Ces complexes ou groupes d’espèces ont des répartitions, des comportements et une écologie différents [3., 4.].

La transmission du paludisme en Afrique subsaharienne est principalement assurée par le complexe *Anopheles gambiae* [2.]. Ce complexe comprend environ neuf sous-espèces apparentées et morphologiquement indiscernables : *Anopheles gambiae*, *Anopheles coluzzii*, *Anopheles arabiensis*, *Anopheles melas*, *Anopheles merus*, *Anopheles bwambae*, *Anopheles quadriannulatus*, *Anopheles amharicus* et *Anopheles fontaneilli* [5.].

La distribution et la densité de ces espèces sont clairement influencées par les conditions environnementales et les caractéristiques climatiques de la région [6.]. *Anopheles arabiensis* prédomine dans les zones les plus sèches. Bien qu’il soit anthropophile, il a fortement tendance à être zoophile. Cependant, en raison de l’évolution constante de l’environnement humain et de l’expansion des villes africaines, *Anopheles arabiensis* est de plus en plus présent dans les grandes villes [7., 8.]. *Anopheles melas* et *Anopheles merus* sont également des vecteurs importants [1., 9.]. *Anopheles melas* est limité aux zones côtières de l’Afrique occidentale et centrale, où ses larves se développent dans les eaux saumâtres [10.]. Cependant, cette espèce n’est pas anthropophile et sa durée de vie est courte, ce qui en fait un mauvais vecteur du paludisme [11.]. *Anopheles merus* est isolé dans la région côtière de l’Afrique de l’Est [1.]. *Anopheles gambiae* et *Anopheles coluzzii* sont sympatriques dans une grande partie, mais pas dans la totalité, de leur aire de répartition en Afrique [12.]. *Anopheles coluzzii* est généralement associé à des sites de reproduction permanents, souvent créés par des activités humaines telles que l’irrigation, la riziculture et l’urbanisation. En revanche, *Anopheles gambiae* est associé à des mares et des flaques d’eau temporaires [13., 14.]. Il n’existe aucune preuve solide que les autres espèces du complexe *An. gambiae* jouent un rôle dans la transmission du paludisme [15., 16.].

En République démocratique du Congo (RDC), le complexe *Anopheles gambiae* joue un rôle majeur dans la transmission du paludisme [17.]. Plus précisément, la plupart des publications font état d’une nette prédominance de l’ancienne forme ss, actuellement reconnue comme *Anopheles gambiae* [18.–21.]. *Anopheles gambiae* est largement distribué à Kinshasa [18., 20.] ; cependant, l’étude de Bobanga, menée en 2013, a noté la présence de l’espèce sympatrique *Anopheles coluzzii* dans le district du Mont Amba à Kinshasa [22.].

Malgré la grande diversité écologique et l’invasion régulière des membres du complexe dans la ville de Kinshasa, aucune étude actualisée sur la composition et la distribution des vecteurs du complexe *An. gambiae* dans cette mégalopole n’est disponible. Cependant, l’identification des espèces au sein du complexe *An. gambiae* est essentielle pour une évaluation correcte de l’écologie des vecteurs du paludisme et des programmes de contrôle [23.]. En outre, il est important de comprendre la structure génétique des membres du complexe *An. gambiae* pour aborder d’importantes questions biologiques et de santé publique, telles que l’évolution, la propagation des allèles de résistance aux insecticides et l’épidémiologie des maladies à transmission vectorielle [24.].

Cette étude vise à établir la composition et la distribution des espèces cryptiques du principal vecteur du paludisme, le complexe *Anopheles gambiae*, circulant dans différents quartiers de la ville de Kinshasa.

## Méthodes

### Couverture spatiale générale

Cette étude a été menée à Kinshasa (RD. Congo) pendant 12 mois, incluant la saison sèche et la saison des pluies (juillet 2021–juin 2022). La ville de Kinshasa est située en Afrique centrale, sur la rive droite du fleuve Congo, entre 4°19′30′′ de latitude sud et 15°19′20′′ de longitude est. Le climat est chaud et humide (AW4 selon la classification de Koppen), avec une saison des pluies d’octobre à mai et une saison sèche de mai à septembre [25.].

La ville de Kinshasa est une mégapole de 9 965 km^2^. Elle est administrativement subdivisée en quatre districts : Tshangu, Funa, Mont Amba et Lukunga. Chaque district est subdivisé en communes et les communes sont subdivisées en quartiers. La transmission du paludisme est pérenne, avec trois strates de transmission [26.]. La strate à faible risque de transmission est située dans le centre-nord de Kinshasa (prévalence du paludisme ≤ 5 %) ; la strate à risque de transmission intermédiaire est située dans le centre-sud de Kinshasa (prévalence du paludisme entre 5 % et 30 %) ; enfin, la strate à risque de transmission élevé est située dans les parties sud-ouest et est de la ville de Kinshasa (prévalence du paludisme > 30 %).

**Figure 1.** Cartes de la ville de Kinshasa montrant les points d’échantillonnage des moustiques *Anophèles*.

 Pour étudier la distribution des membres du complexe *An. gambiae*, les *Anopheles* spp. ont été échantillonnés dans les trois strates de transmission du paludisme dans la ville de Kinshasa [26.]. Dans chaque strate, deux sites (ou quartiers) de deux communes différentes ont été sélectionnés. La sélection de ces sites (Figure 1) a été basée sur la distribution administrative de cette mégapole, en se concentrant sur ses quatre districts :

- District de Funa : Le site de Kimbangu (S 04°20′51′′S, E 15°19′12′′) dans la commune de Kalamu et le site de Valée de Funa (S 04°24′49′′S, E 15°16′52′′) dans la commune de Selembao. Ces deux sites sont situés dans la partie centre-nord de la ville. Ils présentent un risque variable de transmission du paludisme (prévalence ≤ 5 % pour la commune de Kalamu et entre 5 % et 30 % pour la commune de Selembao) [26.] ;
- District du Mont Amba : Le site de Ngwele (S 04°20′59′′, E 15°20′17′′) dans la commune de Limete et le site de Mbanza Lemba (S 4°24′59′′; E 15°19′27′′) dans la commune de Lemba. Ces sites présentent un risque intermédiaire de transmission du paludisme (prévalence entre 5 % et 30 %) [26.] ;
- District de Lukunga : Le site de Djelo Binza (S 04°24′47′′, E 15°20′47′′) dans la commune de Ngaliema et le site de Kitambo (S 04°19′37′′, E 15°16′22′′) dans la commune de Kitambo. Ces deux sites sont situés dans la partie ouest de la ville et présentent un risque intermédiaire de transmission du paludisme (prévalence entre 5 % et 30 %) [26.] ;
- District de Tshangu : Le site de Kikimi (S 4°24′51′′; E 15°24′48′′) dans la commune de Kimbanseke et le site de Ndjii Brasserie (S 4°28′35′′; E 15°21′14′′) dans la commune de Nsele. Ces deux sites sont situés dans la partie Est de la ville et présentent un risque élevé de transmission du paludisme (prévalence > 30 %) [26.].

Dans chaque site d’étude, l’échantillonnage des moustiques a été réalisé à l’aide de pièges lumineux du Center for Disease Control (CDC).

### Capture par piège lumineux CDC

Dans chaque site, dix ménages ont été sélectionnés (avec l’accord des chefs de famille) pour la collecte de moustiques adultes à l’aide de pièges lumineux CDC. Cette collecte a été effectuée la nuit entre 18h00 et 06h00, une fois par mois pendant toute la durée de l’étude. Deux pièges ont été utilisés dans chaque ménage, l’un placé à l’intérieur et l’autre à l’extérieur. Les pièges extérieurs ont été placés à moins de cinq mètres de la porte d’entrée. Pour chaque point de capture, la température et l’humidité ont été mesurées à l’aide de thermohygromètres ThermoPro (ThermoPro, Lawrenceville, USA). Afin d’optimiser la diversité génétique des espèces de Culicidae collectées dans chaque site d’étude, les ménages sélectionnés pour capturer des Culicidae à l’aide de pièges lumineux CDC ont été échantillonnés le long d’un transect de 15 km de long. La répartition des dix ménages sélectionnés par site a suivi le schéma suivant : trois ménages entre 0 et 3 km, trois entre 6 et 9 km et quatre entre 12 et 15 km. Des maisons différentes ont été utilisées pour chaque nuit, avec l’accord des chefs de famille. Les Culicidae capturés ont été envoyés au Laboratoire de Bioécologie et de Lutte Antivectorielle de l’Ecole de Santé Publique de Kinshasa pour une identification morphologique à l’aide de la clé de Coetzee [27.].

### Identification moléculaire des échantillons collectés

Après l’identification morphologique, une proportion égale du complexe *Anopheles gambiae*, échantillonné par site, en fonction de l’étendue du transect, a été conservée individuellement dans des tubes Eppendorf de 1,5 ml avec des gels de silice. Les échantillons ont ensuite été envoyés au Centre de recherche entomologique de Cotonou, au Bénin, pour identifier les espèces cryptiques au sein du complexe *An. gambiae* à l’aide de la réaction en chaîne de la polymérase (PCR).

Après extraction de l’ADN génomique, l’amplification et la détermination de l’espèce du complexe *An. gambiae* ont été réalisées selon le protocole décrit par Scott *et al.* [28.]. À cette fin, les amorces suivantes ont été utilisées : UN: GTGTGCCGCTTCCTCGATGT; AG: CTGGTTTGGTCGGCACGTTT; AA: AAGTGTCCTTCTCCATCCTA; ME: TGACCAACCCACTCCCTTGA; QD: CAGACCAAGATGGTTAGTAT.

L’amorce UN se lie à la même position dans l’ADNr des cinq espèces, tandis que AG se lie spécifiquement à *Anopheles gambiae*. ME se lie à la fois à *Anopheles merus* et à *Anopheles melas*. Enfin, AA se lie à *Anopheles arabiensis* et QD à *Anopheles quadriannulatus*.

Le protocole Santolamazza permet une meilleure différenciation des formes moléculaires de *Anopheles gambiae*, en s’appuyant sur l’insertion spécifique et irréversible d’un transposon de 230 pb (SINE200). Ce transposon est présent sur le chromosome X de *Anopheles coluzzii*, alors qu’il est absent chez *Anopheles gambiae*. Cette caractéristique génétiquement héritée permet la reconnaissance directe, simple et sans ambiguïté de *Anopheles gambiae* et de *Anopheles coluzzii* [29.]. Outre les composants PCR classiques, les amorces suivantes ont été utilisées :

200X6.1F-TCGCCTTAGACCTTGCGTTA and 200X6.1R-CGCTTCAAGAATTCGAGATAC

### Analyse des données

Les données ont été analysées à l’aide du logiciel Origin version 6.1 - Scientific Graphing and Data Analysis Software [30.]. Les relations entre les densités moyennes ont été analysées à l’aide du test *t*-student. Le test de signification a été effectué avec un niveau de signification fixé à 0,05. Une valeur p inférieure à 0,05 a été considérée comme significative au cours de l’analyse. Le diagnostic moléculaire de l’espèce était basé sur le jeu d’amorces S200 X6.1 chez *Anopheles gambiae/coluzzii*, forme hybride (Ac, *Anopheles coluzzii* (479 pb) ; Ag, *Anopheles gambiae* (249 pb) ; nc, contrôle négatif ; L, échelle = 100 pb (Solis BioDyne).

## Résultats

### Composition de la faune d’anophèles spp. à Kinshasa

Durant toute la période de notre étude, 3 839 anophèles ont été collectés à l’aide de pièges lumineux CDC. Après identification morphologique, quatre espèces du genre Anopheles ont été identifiées : *Anopheles gambiae* complex (11,04 %), *Anopheles funestus* gpe (0,53 %), *Anopheles paludis* (1,01 %) et *Anopheles nili* complex (0,13 %) (Figure 2).

**Figure 2.** Distribution des espèces de *Anophèles* de Kinshasa capturées par la technique de piège lumineux CDC au cours de notre étude.

 Alors que le complexe *Anopheles gambiae* a été identifié dans tous les sites, *Anopheles nili* et *Anopheles paludis* ont été limités au district de Tshangu (Tableau 1). A l’exception de *Anopheles paludis*, qui a été capturé plus souvent à l’extérieur des maisons, les autres espèces d’anophèles ont été principalement capturées à l’intérieur des maisons échantillonnées. Par rapport aux autres espèces d’anophèles identifiées, une différence significative de densité moyenne a été observée entre le complexe *Anopheles gambiae* capturé par le piège lumineux CDC à l’intérieur (160,33 ± 40,41) et à l’extérieur (116,50 ± 20,17) des maisons à Kinshasa (*t* =3,36 ; *p* =0,002).

**Tableau 1:** Distribution des espèces anophélienne capturées par la technique de piège lumineux CDC dans les quatre districts de Kinshasa de juillet 2021 à juin 2022.

### Dynamique mensuelle du complexe *Anopheles gambiae* échantillonné à Kinshasa

La dynamique de la densité anophélienne a été variable d’un mois à l’autre au cours de la période d’étude. La densité des moustiques anophèles capturés par les pièges lumineux CDC était très élevée en novembre (10,73 %) et en avril (10,24 %), alors que des densités plus faibles ont été observées en juillet (6,69 %) et en février (6,20 %). La proportion mensuelle du complexe *Anopheles gambiae* capturé au cours de notre étude était élevée en novembre (9,35 %) et en avril (9,20 %). Le pic d’autres espèces anophélienne, distinctes du complexe *Anopheles gambiae*, a été noté en janvier lorsque 1,67 % de tous les *Anopheles* spp. ont été capturés (Figure 3).

**Figure 3.** Distribution mensuelle du complexe *Anopheles gambiae* capturé par la lumière CDC au cours de notre étude.

### Identification moléculaire du complexe *Anopheles gambiae*

Un total de 100 spécimens du complexe *Anopheles gambiae* par site, capturés par la lumière CDC, a été sélectionné. Les résultats de l’identification moléculaire des différentes espèces du complexe *Anopheles gambiae* sont présentés dans le Tableau 2. Deux espèces de ce complexe circulent à Kinshasa : *Anopheles gambiae* et *Anopheles coluzzii*. L’amplification et la détermination des membres du complexe *Anopheles gambiae* ont été réalisées en utilisant une combinaison des protocoles de Scott et de Santolamazza. Les migrations présentées dans la Figure 4 ont permis de distinguer *Anopheles gambiae* (249 pb) et *Anopheles coluzzii* (479 pb) [31.]. Dans tous les sites d’étude, *Anopheles gambiae* était l’espèce principale, avec 674 spécimens sur les 800 du complexe *Anopheles gambiae* passés à l’identification moléculaire (Figure 5).

**Figure 4.** Carte interactive des occurrences géoréférencées hébergées par GBIF [31.]. https://www.gbif.org/dataset/8662eca7-981d-4e75-8ed4-d957644b20bc

**Figure 5.** Image de gel de l’amplification PCR de membres appartenant au complexe *Anopheles gambiae*. Légende: Ac, *Anopheles coluzzii*; Ag, *Anopheles gambiae*; nc, contrôle négatif ; L, échelle.

**Tableau 2:** Identification moléculaire d’espèces cryptiques de *Anopheles gambiae* sl circulant à Kinshasa.

## Possibilité de réutilisation

L’identification des espèces de vecteurs du paludisme circulant dans un environnement donné est essentielle pour une bonne évaluation des programmes de lutte. Quatre espèces du genre *Anopheles* ont été identifiées à travers la ville provinciale de Kinshasa entre juillet 2021 et juin 2022. Il s’agit de *Anopheles gambiae* complex, *Anopheles funestus* gpe, *Anopheles paludis* et *Anopheles nili*. Parmi tous les Anophelinae, le complexe *Anopheles gambiae* était l’espèce la plus importante, suivi par *Anopheles paludis* et *Anopheles funestus* gpe. Ces résultats sont cohérents avec les conclusions d’une étude précédente de Karch *et al.* [32.], qui a trouvé une forte prédominance de *Anopheles gambiae* sl parmi les Anophèles circulant à Kinshasa. Riveron *et al.* en 2018 [18.] et Zanga *et al.* en 2022 [20.], travaillant respectivement dans l’est de la ville de Kinshasa et sur sept sites répartis dans la ville de Kinshasa, ont révélé une nette prédominance du complexe *Anopheles gambiae*. En suivant la dynamique mensuelle du genre *Anopheles*, nous avons montré une fluctuation de leurs fréquences respectives au cours de la période d’étude. Par rapport aux autres espèces qui n’ont pas montré de fluctuations significatives, les fréquences du complexe *Anopheles gambiae* ont augmenté rapidement pendant la saison des pluies, avec des densités maximales enregistrées en novembre et avril, puis ont diminué régulièrement pendant la saison sèche, avec des densités plus faibles en juin et juillet. La fluctuation des précipitations entre les saisons sèches et humides à Kinshasa, associée à la présence de flaques temporaires (mares peu profondes et ensoleillées) largement répandues à Kinshasa pendant la saison des pluies [20.], a été corrélée aux fortes densités du complexe *Anopheles gambiae* [33., 34.].

L’identification moléculaire des spécimens du complexe *Anopheles gambiae* capturés par piège lumineux CDC a montré que *Anopheles gambiae* et *Anopheles coluzzii* sont les seules espèces du complexe *An. gambiae* circulant à Kinshasa. A Kinshasa, ces deux espèces ont été précédemment identifiées par Riveron *et al.* et Zanga *et al.* [18., 20.].

*Anopheles gambiae* a été trouvé dans la plupart des districts de Kinshasa et des sites d’étude sélectionnés. En dehors du district de Lukunga, *An. coluzzii* était présent dans toute la ville de Kinshasa. Sa présence était très élevée dans le district de Tshangu (sites de Kikimi et de Ndjili Brasserie) par rapport aux sites de Mbanza Lemba et de Valée de Funa dans les districts de Mont Amba et de Funa. *Anopheles coluzzii* a également été trouvé dans des proportions élevées sur le site de Ngwele. En 2018, Riveron *et al.* ont également rapporté la coexistence de ces deux espèces sympatriques à Kinshasa, plus précisément à N’Djili Brasserie, dans le district de Tshangu.

La distribution des espèces impliquées dans la transmission du paludisme identifiées dans cette étude constitue une base importante pour mieux définir et orienter les mesures de lutte antivectorielle dans cette mégalopole. Toutefois, il reste essentiel de comprendre l’influence des caractéristiques écologiques sur la distribution de ces espèces et de déterminer l’infectivité de ces vecteurs. Il convient donc d’envisager des études plus approfondies sur cette question.

## Validation des données et contrôle qualité

Les moustiques ont été identifiés par des taxonomistes expérimentés à l’aide d’une clé morphologique bien établie [27.] et de techniques moléculaires [28., 29.]. L’ensemble de données final a été validé dans la boîte à outils de publication intégrée (IPT) du Fonds mondial d’information sur la biodiversité (GBIF) [35.]. L’IPT assure la validation des données par l’intermédiaire de son réseau. Les champs de métadonnées sont disponibles sur la page de l’ensemble de données dans le GBIF [31.].

## Data Availability

Les données utilisées dans cet article ont été publiées par le biais de la boîte à outils de publication intégrée du GBIF [35.] et sont disponibles sous une dérogation CC0 du GBIF [31.].

## Note du rédacteur en chef

Cet article fait partie d’une série d’articles sur la diffusion des données qui travaillent avec le GBIF et sont soutenus par le TDR, le Programme spécial de recherche et de formation sur les maladies tropicales hébergé par l’Organisation mondiale de la santé [36.].

## Abréviations

CDC, Centre de contrôle des maladies ; RDC, République démocratique du Congo ; GBIF, Fonds mondial d’information sur la biodiversité ; IPT, boîte à outils de publication intégrée ; PCR, réaction en chaîne de la polymérase.

## Déclarations

### Approbation éthique et consentement à la participation

Les auteurs déclarent que l’approbation éthique n’était pas nécessaire pour ce type de recherche.

### Informations sur les auteurs

JZ, NM, VN, OM et MB sont impliqués dans la surveillance et le contrôle des vecteurs, ainsi que dans des études de durabilité des moustiquaires imprégnées d’insecticide au niveau national, et sont tous des chercheurs au sein de l’unité de Bioécologie et lutte antivectorielle de l’école de santé publique de Kinshasa (Université de Kinshasa). EM et FA mènent des activités de cartographie des vecteurs et de surveillance de la résistance au niveau national, et sont tous des chercheurs de l’unité d’entomologie de l’Institut national de recherche biomédicale. FA est également le chef d’équipe du projet PMI Evolve en RDC et chercheur au Centre de Recherche Entomologique de Cotonou (CREC), Cotonou, Bénin. Pendant cette étude, EM était également professeur à l’Université de Bandundu, Faculté de médecine, Bandundu Ville, République démocratique du Congo. NB mène des études sur la cartographie des Anophèles vecteurs du paludisme au niveau national et sur la surveillance des espèces envahissantes. DB et RW sont chercheurs en entomologie à l’Université de Kinshasa, département de neurologie, Kinshasa, République démocratique du Congo.

### Financement

Le matériel et le soutien financier pour cette étude ont été fournis par l’unité de Bioécologie et de lutte antivectorielle du département de santé et environnement de l’Ecole de Santé Publique de Kinshasa. Aucun financement externe ou soutien financier n’a été obtenu.

## References

[1] WiebeA, LongbottomJ, GleaveK Geographical distributions of African malaria vector sibling species and evidence for insecticide resistance. Malar. J., 2017; 16: 85. doi:10.1186/s12936-017-1734-y.28219387 PMC5319841

[2] ServiceMW, TownsonH. The *Anopheles* Vector. In: GillesHM, WarrellDA (eds), Essential Malariology. 4th ed., London: Arnold, 2002; p. 59, doi:10.1201/9780203756621.

[3] HaySI, SinkaME, OkaraRM Developing global maps of the dominant *Anopheles* vectors of human malaria. PLoS Med., 2010; 7(2): e1000209. doi:10.1371/journal.pmed.1000209.20161718 PMC2817710

[4] SinkaME, BangsMJ, ManguinS A global map of dominant malaria vectors. Parasites Vectors, 2012; 5: 69. doi:10.1186/1756-3305-5-69.22475528 PMC3349467

[5] BarrónMG, PaupyC, RaholaN A new species in the major malaria vector complex sheds light on reticulated species evolution. Sci. Rep., 2009; 9: 14753. doi:10.1038/s41598-019-49065-5.PMC679187531611571

[6] OyewoleSO, IbidapoAC, OduolaAO Molecular identification and population dynamics of the major malaria vectors in a rain forest zone of Nigeria. Biokemistri, 2005; 17(2): 171–178.

[7] JonesCM, ToeHK, SanouA Additional selection for insecticide resistance in Urban Malaria vectors: DDT resistance in *Anopheles arabiensis* from Bobo-Dioulasso, Burkina Faso. PLoS One, 2012; 7(9): e45995. doi:10.1371/journal.pone.0045995.23049917 PMC3457957

[8] DabiréRK, NamountougouM, SawadogoSP Population dynamics of *Anopheles gambiae* s.l. in Bobo-Dioulasso city: bionomics, infection rate and susceptibility to insecticides. Parasites Vectors, 2012; 5: 127. doi:10.1186/1756-3305-5-127.22721002 PMC3424103

[9] TunoN, KjaerandsenJ, BaduK Blood-feeding behavior of *Anopheles gambiae* and *Anopheles melas* in Ghana, western Africa. J. Med. Entomol., 2010; 47(1): 28–31. doi:10.1603/033.047.0104.20180305

[10] DiagneN, FontenilleD, KonatéL Les anophèles du Sénégal. Bull. Soc. Pathol. Exot., 1994; 87: 267–277.7866049

[11] LemassonJ, FontenilleD, LochouarnL Comparison of behaviours and vector efficiency of *Anopheles gambiae* and *Anopheles arabiensis* (Diptera, Culicidae) in Barkedji, a sahelian area of Senegal. J. Med. Entomol., 1997; 34: 396–403. doi:10.1093/jmedent/34.4.396.9220672

[12] WondjiC, SimardF, FontenilleD. Evidence for genetic differentiation between the molecular forms M and S within the Forest chromosomal form of *Anopheles gambiae* in an area of sympatry. Insect. Mol. Biol., 2002; 11(1): 11–19. doi:10.1046/j.0962-1075.2001.00306.x.11841498

[13] SimardF, AyalaD, KamdemGC Ecological niche partitioning between *Anopheles gambiae* molecular forms in Cameroon: the ecological side of speciation. BMC Ecol., 2009; 9: 17. doi:10.1186/1472-6785-9-17.19460146 PMC2698860

[14] CostantiniC, AyalaD, GuelbeogoWM Living at the edge: biogeographic patterns of habitat segregation conform to speciation by niche expansion in *Anopheles gambiae* . BMC Ecol., 2009; 9: 16. doi:10.1186/1472-6785-9-16.19460144 PMC2702294

[15] KipyabPC, KhaembaBM, MwangangiJM The bionomics of *Anopheles merus* (Diptera: Culicidae) along the Kenyan coast. Parasites Vectors, 2013; 6: 37, doi:10.1186/1756-3305-6-37.23410133 PMC3584914

[16] EbenezerA, NoutchaAEM, OkiweluSN. Relationship of annual entomological inoculation rates to malaria transmission indices, Bayelsa State, Nigeria. J. Vector Borne Dis., 2016; 53: 46–53.27004578

[17] National Strategic Plan for Malaria Control 2016–2018. Malaria Control Program. Kinshasa, DR Congo. 2016.

[18] RiveronJ, WatsengaF, IrvingH High plasmodium infection rate and reduced bed net efficacy in multiple insecticide-resistant malaria vectors in Kinshasa, Democratic Republic of Congo. J. Infect. Dis., 2018; 217(2): 320–328. doi:10.1093/infdis/jix570.29087484 PMC5853898

[19] Metelo-MatubiE, ZangaJ, BineneG The effect of a mass distribution of insecticide-treated nets on insecticide resistance and entomological inoculation rates of *Anopheles gambiae* s.l. in Bandundu City, Democratic Republic of Congo. Pan. Afr. Med. J., 2021; 40: 118. doi:10.11604/pamj.2021.40.118.27365.34887992 PMC8627145

[20] ZangaJ, MeteloE, MbanzuluK Susceptibility status of *Anopheles gambiae* s.l to insecticides used for malaria control in Kinshasa, Democratic Republic of the Congo. Ann. Afr. Med., 2022; 15(2): e4533–e4542.

[21] BandibaboneJ, McLoughlinC, N’DoS Investigating molecular mechanisms of insecticide resistance in the Eastern Democratic Republic of the Congo. Malar. J., 2021; 20(1): 464. doi:10.1186/s12936-021-04002-8.34906124 PMC8670120

[22] BobangaT, AyiekoW, ZangaM Field efficacy and acceptability of PermaNet^®^ 3.0 and OlysetNet^®^ in Kinshasa, Democratic Republic of the Congo. J. Vector Borne Dis., 2013; 50(3): 206–214.24220080

[23] FanelloC, PetrarcaV, della TorreA The pyrethroid knock-down resistance gene in the *Anopheles gambiae* complex in Mali and further indication of incipient speciation within *An. gambiae* s.s. Insect. Mol. Biol., 2003; 12(3): 241–245. doi:10.1046/j.1365-2583.2003.00407.x.12752657

[24] GaleKR, CramptonJM. DNA probes for species identification of mosquitoes in the Anopheles gambiae complex. Med. Vet. Entomol., 1987; 1(2): 127–136. doi:10.1111/j.1365-2915.1987.tb00333.x.2979526

[25] KottekM, GreiserJ, BeckC World Map of the Köppen-Geiger climate classification updated. Meteorol. Z., 2006; 15: 259–263. doi:10.1127/0941-2948/2006/0130.

[26] FerrariG, NtukuH, SchmidlinS A malaria risk map of Kinshasa, Democratic Republic of Congo. Malar. J., 2016; 15: 27. doi:10.1186/s12936-015-1074-8.26762532 PMC4712518

[27] GilliesMT, CoetzeeM. A supplement to the Anophelinae of Africa south of the Sahara (Afrotropical Region). Johannesburg: South African Institute for Medical Research, 1987; pp. 1–143, OCLC: 1408893217.

[28] ScottJA, BrogdonWG, CollinsFH. Identification of single specimens of the *Anopheles gambiae* complex by the polymerase chain reaction. Am. J. Trop. Med. Hyg., 1993; 49: 520–529. doi:10.4269/ajtmh.1993.49.520.8214283

[29] SantolamazzaF, ManciniE, SimardF Insertion polymorphisms of SINE200 retrotransposons within speciation islands of *Anopheles gambiae* molecular forms. Malar. J., 2008; 7: 163. doi:10.1186/1475-2875-7-163.18724871 PMC2546427

[30] Origin version 6.1-scientific graphing and data analysis software. https://www.originlab.com/index.aspx?go=Support&pid=468.

[31] ZangaJ, MeteloE, MvuamaN Species composition and distribution of *Anopheles gambiae* complex circulating in Kinshasa. Version 1.2. University of Kinshasa. 2023. Sampling event dataset 10.15468/excax3 accessed via GBIF.org on 2023-10-24.

[32] KarchS, AsidiN, ManzambiZ La faune culicidienne et sa nuisance à Kinshasa (Zaïre). Bull. Soc. Pathol. Exot., 1993; 86: 68–75.8504267

[33] MeteloME, BukakaE, BakambanaT Détermination des paramètres bioécologiques et entomologiques de *Anopheles gambiae* sl dans la transmission du paludisme à Bandundu-ville, RD Congo. Pan. Afr. Med. J., 2015; 22: 108. doi:10.11604/pamj.2015.22.108.6774.26848355 PMC4732634

[34] SougoufaraS, DoucouréS, SembénePM Challenges for malaria vector control in sub-Saharan Africa: resistance and behavioral adaptations in Anopheles populations. J. Vector Borne Dis., 2017; 54: 4–15.28352041

[35] Global Biodiversity Information Facility (GBIF). IPT: The Integrated Publishing Toolkit. https://www.gbif.org/en/ipt. Accessed 1 September 2023.

[36] Vectors of Human Disease Series. *GigaByte*. 2022; 10.46471/GIGABYTE_SERIES_0002.
